# Choroidal Structural Changes of Posterior Subtenon Triamcinolone Acetonide Injection in Eyes with Refractory Diabetic Macular Edema

**DOI:** 10.1155/2022/6882607

**Published:** 2022-02-21

**Authors:** Bing Liu, Guangfeng Ma, Jing Hou, Chenyang Cong

**Affiliations:** ^1^Department of Ophthalmology, The Second Hospital, Cheeloo College of Medicine, Shandong University, Jinan 250033, China; ^2^Affiliated Eye Hospital of Shandong University of Traditional Chinese Medicine, Jinan 250002, China

## Abstract

**Purpose:**

To access the choroidal structural changes of posterior subtenon triamcinolone acetonide (PSTA) injection in eyes with refractory diabetic macular edema (DME).

**Methods:**

Patients with refractory DME were enrolled and followed for 4 weeks after switching to PSTA injection. All patients underwent spectral-domain optical coherence tomography with enhanced depth imaging, and the choroidal images were binarized into the luminal area and total choroidal area. Subfoveal choroidal thickness (SFCT) and choroidal vascularity index (CVI) were evaluated before and after switching treatments.

**Results:**

After switching to PSTA treatment, the final best-corrected visual acuity and central subfield thickness in eyes with refractory DME were significantly improved compared to the baseline values (*P*=0.002 and *P* < 0.001, respectively). Both the SFCT and CVI decreased during the follow-up period, and significant decreases were observed at 4-week follow-up (*P* < 0.001 and *P*=0.012, respectively). The linear regression analysis showed a significant correlation between the baseline SFCT and the final visual outcomes (*P*=0.047).

**Conclusions:**

The alterations of SFCT and CVI in this study suggest that the choroidal vasculature is involved in the pathogenesis of refractory DME and could be affected by PSTA treatment. SFCT rather than CVI may be a prognostic biomarker for eyes with refractory DME.

## 1. Introduction

Diabetic macular edema (DME) is the leading cause of vision loss in patients with diabetic retinopathy (DR) [1]. Treatments for DME include anti-vascular endothelial growth factor (VEGF) agents, laser photocoagulation, steroids, and vitrectomy. Due to its significant outcomes, intravitreal injection of anti-VEGF agents has been generally accepted as the first-line therapy for DME worldwide [2–4]. However, not all patients with DME respond to anti-VEGF treatment. More than 40% of DME patients still have thickened central macular thickness even after numerous intravitreal injections [5]. The pathogenesis of DME is multifactorial and complex. Both VEGF and other inflammatory mediators are involved in it [6]. If the response to anti-VEGF therapy is inadequate, switching to alternative treatment modalities such as steroids should be considered [2, 4].

The choroid plays an important role in DR, and the changes in the choroidal structure could be used as an indicator of the effectiveness of treatment. Previous studies have reported that the subfoveal choroid was thicker in eyes with DME than in those without DME and the subfoveal choroidal thickness (SFCT) became thinner after treatment TA in eyes with DME [7, 8]. However, choroidal thickness alone is only partially representative of its overall structural changes. Recently, the choroidal vascularity index (CVI) has been widely used to assess the vascular status of the choroid in many ocular diseases [9]. Unlike choroidal thickness, CVI is not affected by physiological and ocular factors and CVI also shows less variability among healthy eyes [10]. With the introduction of CVI, researchers can evaluate the choroid more comprehensively.

Triamcinolone acetonide (TA), as an alternative drug for cases not responding to anti-VEGF treatment, has been widely used in clinical ophthalmology [11–13]. Although many studies on TA in the treatment of DME had been performed in the past, most of them focused on the efficacy and safety of it. To the best of our knowledge, no published study has previously evaluated the effect of posterior subtenon TA (PSTA) injection on the choroidal structure in eyes with DME resistant to anti-VEGF agents.

Thus, the purpose of this study was to investigate the choroidal structural changes of PSTA injection in eyes with refractory DME.

## 2. Materials and Methods

A retrospective review was performed on patients with refractory DME who were referred to the Department of Ophthalmology at the Second Hospital of Shandong University in Jinan between January 2019 and February 2021. The study was performed in accordance with the provisions of the Declaration of Helsinki and was approved by the Ethical Review Committee of the Second Hospital of Shandong University. All patients were informed about the potential side effects, and their consent was taken before the procedure.

The inclusion criteria for patients with refractory DME were as follows: (1) age >18 years, (2) type 2 diabetes mellitus and nonproliferative DR, and (3) the refractive errors ranging from −3.0 diopters to +1.5 diopters. Refractory DME was defined as central subfield thickness (CST) still >300 mm or change in CST <10% after 3 consecutive anti-VEGF injections. Patients with the following conditions were excluded: (1) a history of other ocular disorders, such as significant cataract, glaucoma, or any other associated retinal vascular diseases; (2) previous focal or grid laser treatment; (3) panretinal photocoagulation (PRP) treatment for <3 months; (4) previous administration of TA; and (5) a history of any intraocular surgery.

PSTA injection was performed using the same protocol. After topical anesthesia of the conjunctiva with drops of 0.5% proparacaine, the patient was asked to gaze at the superonasal direction. Then, 20 mg of TA was administered inferotemporally into the posterior subtenon space with a 27-gauge needle.

**Figure 1:**
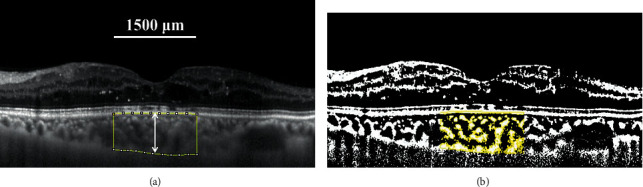
Enhanced depth SD-OCT image and converted binary image of the eye with refractory DME. The prebinarized SD-OCT image of the eye with refractory DME. The region within the dotted lines represent the selected TCA. SFCT (white arrow) is defined as the vertical distance from the outer surface of the retinal pigment epithelium to the choroidal-scleral interface (a). The postbinarized SD-OCT image of the eye with refractory DME (b).

All patients underwent a comprehensive ophthalmic examination at baseline and 1 and 4 weeks after injection, including best-corrected visual acuity (BCVA), intraocular pressure (IOP), slit-lamp biomicroscopy, dilated fundus examination by indirect ophthalmoscopy (+90 D), and CST measured by spectral-domain optical coherence tomography (SD-OCT) with enhanced depth imaging (EDI) (Spectralis HRA + OCT, Heidelberg Engineering, Heidelberg, Germany). All EDI-OCT images were obtained between 11:00 and 13:00 hours to minimize the effect of diurnal variations in the choroidal structures [14]. SFCT from foveal scans was measured vertically from the outer surface of the retinal pigment epithelium to the choroidal-scleral interface (Figure 1(a)).

The CVI was calculated using a previously reported method [15, 16]. The EDI-OCT image was analyzed by Image J software (version 1.51; https://imagej.nih.gov/ij/). The total choroidal area (TCA) with a width of 1500 *μ*m (750 *μ*m on either side of the central fovea) was selected with the polygon tool and added to the region of interest (ROI) manager. Then, three choroidal vessels with large lumens (>100 *µ*m) were selected with the oval selection tool, and the average reflectivity of these areas was measured. After adjusting the image using the average brightness determined above as the minimum value, the image was converted to 8-bit format and Niblack auto-local thresholding was subsequently applied. The LA was highlighted by applying the color threshold and later added to the ROI manager. Then, both areas in the ROI manager were selected and merged into the composite third area by the AND operation. The first area represents the TCA, and the third area represents the LA (Figure 1). Finally, the CVI was calculated by dividing the LA by the TCA.

### 2.1. Statistical Analysis

Data were processed and analyzed using SPSS 21.0 software (Chicago, IL, USA). To evaluate the differences in BCVA, IOP, CST, SFCT, and CVI before and after PSTA treatment, paired *t*-tests were performed. Linear regression analysis was conducted between the baseline choroidal parameters and BCVA measured at 4-week follow-up. A *P* value <0.05 was considered statistically significant.

Statistical power analysis was performed using G^*∗*^Power version 3.1.9.7 (Franz Faul, Universitat Kiel, Germany) with an *α* level of 5%. For the sample size of 21 subjects, the power is 88.6% that the study detects a difference at a two-sided 0.05 significance level, if the effect size is 0.5.

## 3. Results

### 3.1. Baseline Characteristics

A total of 21 eyes of 21 patients with refractory DME were enrolled in this study; all of them received 4 weeks of follow-up examination. The clinical characteristics of them are summarized in Table 1. Of 21 patients, 12 (57.1%) were males and 9 (42.9%) were females. The mean age of the patients was 58.2 ± 7.5 years. The mean duration of diabetes mellitus was 11.6 ± 5.6 years. PRP had been performed in 5 of the 21 eyes (23.8%).

### 3.2. Best-Corrected Visual Acuity

**Figure 2:**
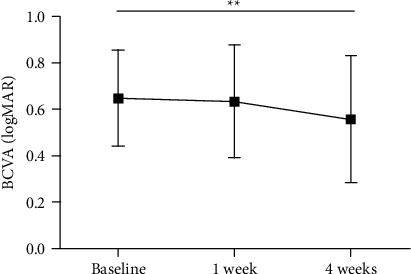
Changes in the BCVA before and after switching to PSTA treatment in eyes with refractory DME. ^*∗∗*^*P* < 0.01*vs.* the baseline value.

As shown in Figure 2, the mean BCVA for all eyes was improved during the follow-up period. Compared with the mean BCVA at the baseline, the mean BCVA improved to 0.63 ± 0.24 logMAR at 1 week (*P*=0.480) and 0.56 ± 0.27 logMAR at 4 weeks (*P*=0.002).

### 3.3. Intraocular Pressure

The mean IOP for all eyes at baseline, 1 week, and 4 weeks was 14.4 ± 3.04 mmHg, 15.1 ± 3.10 mmHg, and 14.9 ± 2.78 mmHg, respectively. During the follow-up period, there was no significant difference in IOP compared to the baseline (*P*=0.429 and *P*=0.539, respectively).

### 3.4. Retinal Thickness

**Figure 3:**
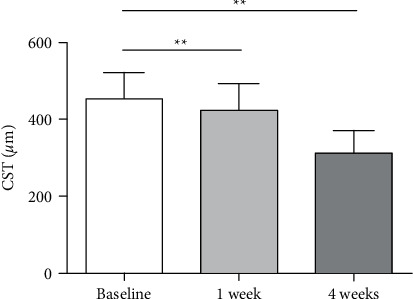
Changes in the CST before and after switching to PSTA treatment in eyes with refractory DME. ^*∗∗*^*P* < 0.01*vs.* the baseline value.

The mean CST for all eyes decreased significantly from 453.4 ± 68.7 *μ*m at baseline to 422.0 ± 70.6 *μ*m at 1 week and 311.9 ± 59.9 *μ*m at 4 weeks (all *P* < 0.001, Figure 3).

### 3.5. Choroid Vascular Characteristics

Table 2 shows the comparisons of choroid vascular characteristics before and after PSTA treatment. The mean SFCT in all eyes with refractory DME decreased from 304.2 ± 59.9 *μ*m at baseline to 300.5 ± 56.4 *μ*m at 1 week (*P*=0.156) and 293.5 ± 58.7 *μ*m at 4 weeks (*P*=0.001). The CVI of eyes with refractory DME was 64.4 ± 5.6 at baseline and decreased to 63.5 ± 6.1 at 1 week (*P*=0.261) and 61.6 ± 5.9 at 4 weeks (*P*=0.012).

### 3.6. Correlation between Baseline Choroidal Parameters and the Final BCVA

**Figure 4:**
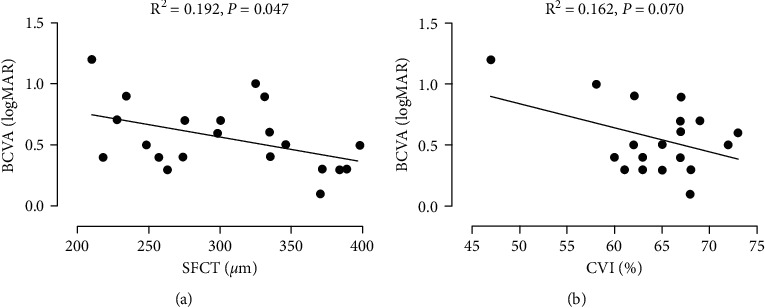
The linear regression analysis between the choroidal parameters and the final BCVA after switching to PSTA treatment.

The results of the linear regression analysis showed a significant correlation between baseline SFCT and BCVA at 4 weeks after switching to PSTA treatment (*P*=0.047, Figure 4(a)). However, no significant correlation was found between baseline CVI and the final BCVA (*P*=0.070, Figure 4(b)).

### 3.7. Systemic/Ocular Complications

No systemic or ocular complications were detected during the follow-up period.

## 4. Discussion

In the present study, we found that PSTA injection could significantly reduce the CST and improve BCVA in DME eyes refractory to anti-VEGF therapy. After switching to PSTA treatment, both SFCT and CVI of eyes with refractory DME were significantly decreased at 4 weeks during the follow-up period. Moreover, a significant correlation was found between the baseline SFCT and the final visual outcomes.

The pathogenesis of DME is complex, involving VEGF and other inflammatory mediators [6]. Compared with anti-VEGF therapy, steroids can reduce not only the levels of VEGF but also those of other inflammatory cytokines [17]. Thus, steroids should be considered for some cases that are refractory to anti-VEGF therapy. TA is commonly used to treat DME in clinical ophthalmology, either by intravitreal injection or by posterior subtenon injection. In a meta-analysis study on the effects of PSTA and intravitreal triamcinolone acetonide (IVTA) on DME eyes, IVTA significantly improved the BCVA at 3 months. However, the benefit was not significant at 6 months, and the IOPs were significantly higher at 3 and 6 months [18]. To avoid the complications of IVTA, PSTA was selected in this study. Furthermore, the sustained-release steroid agent was not used in this study because it has not been approved in the treatment of DME in China. Irreversible photoreceptor damage may occur with persistent DME, and timely management is crucial to prevent vision loss. For cases with resistant DME, early switching from anti-VEGF to steroids offers better treatment outcomes [19]. Thus, the PSTA treatment was performed after 3 anti-VEGF injections in this study.

Management of DME focuses on improving anatomical and functional outcomes, such as reducing CST and improving visual acuity. In this study, CST and BCVA improved after switching to PSTA treatment, which was consistent with previous reports [11–13]. IOP elevation and cataract progression are the most common complications of PSTA, but the incidences of them are very low [20]. No systemic or ocular complications, especially cataract progression, were detected in this study, which may be explained by the low risk of side effects due to PSTA. In addition, the short follow-up period and small sample size in this study may also have contributed to the results.

The choroid is one of the most vascularized structures of the human body and is essential in providing nutrients and oxygenation to the outer retina layers. Disruption of its structure or vasculature can affect the retinal function. Understanding the choroidal vasculature will provide valuable insights into the pathogenesis of DME and the development of treatment strategies. SFCT and CVI are commonly used to access the status of choroid currently. The choroidal thickness in eyes with DME was contradictory in previous studies, but it is generally accepted that the choroidal thickness in eyes with DME becomes thinner after treatment [7, 21–23]. The increased choroidal thickness in eyes with DME is mainly caused by vasodilation and tissue edema, which can be suppressed by steroids. In this study, a decreased SFCT was observed after PSTA treatment, although the difference was not significant at 1 week. After PSTA treatment for 4 weeks, the SFCT decreased significantly, associated with an increase in BCVA, which was consistent with previous studies [7, 22, 23]. Decreased SFCT was also reported in DME eyes after intravitreal dexamethasone implant injection. Compared with PSTA treatment, significant alterations in SFCT could be observed in the early stage of intravitreal dexamethasone implant injection, even after 1 day [23–25]. Furthermore, as a sustained-release steroid agent, the significant effect of dexamethasone implant on SFCT is durable, which can last for 14 weeks [23].

The analysis of choroidal changes using SFCT alone has some limitations because SFCT can be affected by various physiological factors and has exhibited notable disparity in various clinical studies [9, 26]. In this study, we used CVI, a more stable and objective quantitative marker for the assessment of choroidal vascularity, to overcome the limitations associated with using SFCT alone. In this study, we found that both TCA and LA decreased after PSTA treatment, but the differences in TCA were not significant. The same results were also observed in DME eyes after anti-VEGF treatment in the previous study [8]. The decreased TCA was related to the reduction of choroidal thickness. The significantly decreased LA at the final visit indicated that vasodilation caused by inflammation was relieved after PSTA treatment. The decreased vasodilation of the choroid would result in a decrease in CVI. In this study, the CVI significantly decreased at the final visit compared to baseline, which demonstrated that choroidal vascular lumens were exclusively affected rather than the stroma in patients with DME after steroid treatment and the changes of choroidal vascular lumens may play a more important role in the pathogenesis of DME.

Recent studies reported that greater baseline SFCT and CVI were significantly associated with better visual outcomes after intravitreal anti-VEGF injections [27, 28]. In the present study, we found that only SFCT was significantly correlated with BCVA at 4-week follow-up, which indicated that SFCT, rather than CVI, might be a prognostic biomarker for visual response to PSTA treatment in refractory DME.

There are several limitations to our study that need to be considered. First, the number of patients who participated in this study was small. Second, a referral bias may exist because all patients were recruited from a single center. Finally, the follow-up period in this study was short. Therefore, a multicenter clinical study with more patients and a longer follow-up period may be warranted in the future.

In conclusion, switching to PSTA treatment can be a good option for DME eyes refractory to anti-VEGF injections. The alteration of SFCT and CVI suggests that the choroidal vasculature is involved in the pathogenesis of refractory DME and could be affected by PSTA treatment. SFCT rather than CVI may be a prognostic biomarker for eyes with refractory DME.

## Figures and Tables

**Table 1:** Patient clinical characteristics (*n* = 21).

Characteristics	
Age (years)	58.2 ± 7.5
Female/total (*n*, %)	9/21 (42.9)
Duration of diabetes mellitus (years)	11.6 ± 5.6
HbA1c (mg/dl)	6.5 ± 1.0
PRP treated (*n*, %)	5 (23.8)

*Prior anti-VEGF injections (n, %)*	
Ranibizumab	24 (38.1)
Conbercept	33 (52.4)
Aflibercept	6 (9.5)
BCVA (logMAR)	0.65 ± 0.21
IOP (mmHg)	14.4 ± 3.04
CST (*μ*m)	453.4 ± 68.7

**Table 2:** Changes of choroidal parameters in eyes with refractory DME after switching to PSTA treatment.

	Baseline	1 week	1 month
LA (mm^2^)	0.35 ± 0.10	0.34 ± 0.09	0.32 ± 0.09^*∗*^
TCA (mm^2^)	0.55 ± 0.13	0.54 ± 0.14	0.52 ± 0.11
CVI (%)	64.4 ± 5.6	63.5 ± 6.1	61.6 ± 5.9^*∗*^
SFCT (*μ*m)	304.2 ± 59.9	300.5 ± 56.4	293.5 ± 58.7^*∗*^

^*∗*^*P* < 0.05*vs.* the baseline value.

## Data Availability

The data used to support the findings of this study are currently under embargo because the fund projects supporting this study have not yet been concluded. Six months after the publication of this article, the data will be made available from the corresponding author upon request.
